# Ivacaftor modifies cystic fibrosis neutrophil phenotype in subjects with R117H residual function CFTR mutations

**DOI:** 10.1183/13993003.02161-2020

**Published:** 2021-01-21

**Authors:** Gareth R. Hardisty, Sheonagh M. Law, Suzanne Carter, Brenda Grogan, Pradeep K. Singh, Edward F. McKone, Robert D. Gray

**Affiliations:** 1 University of Edinburgh, Centre for Inflammation Research, Edinburgh, UK; 2 St Vincent's Hospital, University College Dublin, Dublin, Ireland; 3 Dept of Microbiology, University of Washington School of Medicine, Seattle, WA, USA

## Abstract

**CFTR modulation leads to changes in neutrophil phenotype even in patients with residual function CFTR mutations** https://bit.ly/2EUk7xH

To the Editor:

Cystic fibrosis transmembrane conductance regulator (CFTR) modulator therapy (ivacaftor, lumacaftor, tezacaftor) treats the basic defect in cystic fibrosis (CF) by increasing CFTR function and improving lung function and quality of life. CF lung disease is characterised by chronic bacterial colonisation, inflammation and excessive neutrophilia [1]. The confirmation of CFTR expression on neutrophils [2] led to speculation that immune cell dysfunction may be implicated in CF lung inflammation. Neutrophils from CF patients with severe CFTR mutations (*e.g.* F508del and G551D) have prolonged neutrophil survival [3] and decreased phagocytosis and degranulation. The residual function R117H mutation causes a 25% decrease in channel conductance [4], and when present in combination with a second severe mutation (*e.g.* F508del) results in CFTR function that lies somewhere between heathy controls and typical CF. CF patients with residual function develop disease at a later stage and ivacaftor is now licensed for the treatment of the R117H mutation having been demonstrated to be effective in clinical trials [5]. Treatment of people with G551D mutations with ivacaftor also has significant mutation specific effects on myeloid cells [6]. Therefore, we assessed the effects of CFTR modulator therapy on neutrophil phenotype and function in this group.

The St Vincent's Healthcare Ethics and Medical Research Committee, Dublin, Ireland, approved the study. 10 clinically stable CF subjects with one copy of the R117H allele and a second disease causing mutation were recruited. Patients received 150 mg ivacaftor twice daily for 7 days. Whole blood was collected before treatment and at day two and seven during treatment. Neutrophils were isolated by Ficoll Paque gradient in SepMate tubes followed by hypotonic red blood cell lysis to reduce potential artefactual activation by dextran sedimentation [7, 8], and flow cytometry was performed according to standard intra- and extracellular staining protocols [9]. Samples were also collected from six healthy controls and four people with CF with class I–III mutations (NRS Bioresource, East of Scotland research ethics committee 15/ES/0094 and AMREC 15-HV-013).

Six male and four female CF subjects (mean±sd age 40.5±7.21 years) were recruited, all having at least one copy of the R117H gene in combination with another disease causing mutation (F508del seven subjects, M156R two subjects, and 2622+1G→A one subject). Three subjects were colonised with *Staphylococcus aureus*, two with *Pseudomonas aeruginosa*, three with a combination of *S. aureus* and *P. aeruginosa*, and two subjects had no colonising organisms. Sweat chloride changed from 77.5±4 mmol·L^−1^ to 52.1±5.61 mmol·L^−1^ after 7 days of treatment (p<0.001, paired t-test) confirming a pharmacodynamic response to therapy; forced expiratory volume in 1 s (FEV_1_) also improved at 7 days, as detailed in a contemporaneous study in the same patient cohort [10].

Treatment with ivacaftor had no significant impact on total isolated granulocyte number (figure 1a) or frequency of circulating neutrophils in peripheral blood (figure 1b). Surprisingly, and even before treatment, R117H neutrophils had similar survival rates to healthy controls at 24 h (figure 1c). This is in contrast to neutrophils from class I–III mutation subjects with CF, where 40% were viable at 24 h. Additionally, there was no change in neutrophil survival following 7 days of ivacaftor treatment, further underlined by stable expression of extrinsic cell death receptors CD95 (FASR), CD120a (TRAIL-receptor 2), and CD120b (tumour necrosis factor receptor) before and after ivacaftor treatment (figure 1e).

**FIGURE 1:**
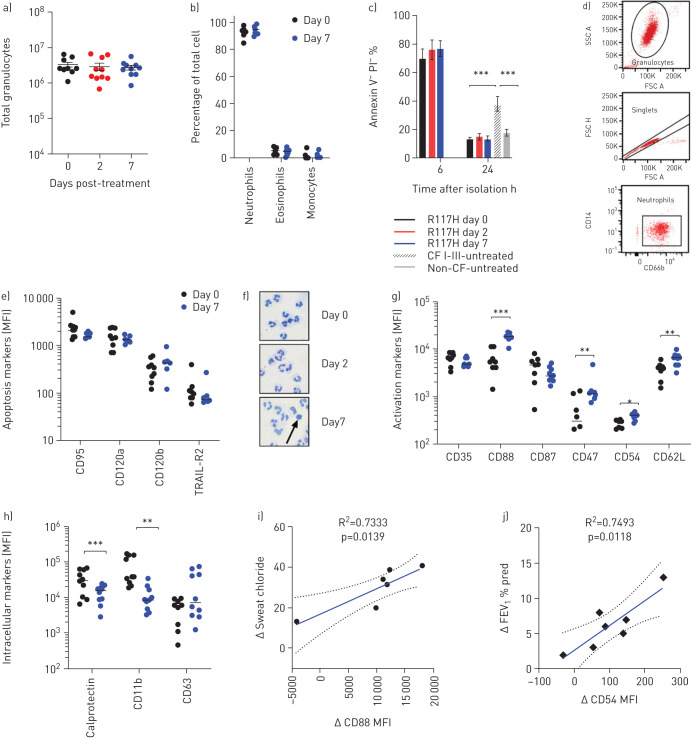
Neutrophils from subjects with R117H mutation show no apoptosis defect but ivacaftor treatment induces changes in activation and adhesion markers after 7 days of therapy. a) Total peripheral granulocytes isolated by Ficoll Paque gradient in SepMate tubes and hypotonic RBC lysis before treatment and following 2 and 7 days of treatment. b) Percentage of granulocyte cell types isolated demonstrate high purity neutrophils. c) Percentage of viable neutrophils, 6 and 24 h after isolation in R117H subjects (pre-treatment and following 2 and 7 days of treatment) compared to untreated cystic fibrosis (CF) with type I–III mutations and non-CF controls (R117H, n=7–10; CFI–III, n=4; non-CF, n=6). d) Gating strategy for neutrophil flow cytometry. Granulocytes were enriched from Ficoll pellet after RBC hypotonic lysis. Neutrophils were gated by FSC×SSC, singlets were identified by FSC-A×FSC-H and neutrophils by CD66b+ CD14- Ab staining. e) Extrinsic cell death receptor expression measured by flow cytometry and shown as mean fluorescence intensity (MFI). f) Morphological structure of isolated neutrophils (10× magnification). Black arrow signifies apoptotic cell. g) Neutrophil activation and adhesion marker expression. Individual markers analysed by t-test. h) Markers of neutrophil granules. Individual markers analysed by t-test. i) Correlation of change in neutrophil CD88 expression (MFI) with change in patient sweat chloride from day 0–7 analysed by linear regression. j) Correlation of change in neutrophil CD54 expression (MFI) with change in patient forced expiratory volume in 1 s (FEV_1_) % predicted. All data are shown as mean±sem. Statistically significant values designated by asterisks. *: p<0.05 **: p<0.01 ***: p<0.001.

Next, we measured multiple surface markers by flow cytometry before and 7 days after ivacaftor treatment. There were statistically significant increases in CD88 (C5a receptor), CD47 (integrin associated protein), CD54 (intracellular adhesion molecule 1) and CD62L (L-selectin) in response to ivacaftor treatment (figure 1g), whereas CD35 and CD87 did not change. We also measured intracellular expression of a number of key neutrophil proteins and found that the cytosolic protein and damage associated molecular pattern (DAMP) calprotectin, and the secondary granule protein activated CD11b decreased with ivacaftor treatment (figure 1h), whereas CD63 (tetraspanin), a primary granule protein, did not change significantly. Increased expression of CD54, CD88 and CD47 on neutrophils were associated with a decrease in patient sweat chloride, although only significantly for CD88 (figure 1i), suggesting a possible association with CFTR function. Only CD54 showed an association with changes in lung function (FEV_1_) after treatment (figure 1j).

Our data demonstrate that CFTR modulator therapy leads to phenotypic changes in neutrophils from CF patients with residual CFTR function. Interestingly a pro-survival neutrophil phenotype, which is well described in CF patients with severe mutations (class I–III) was absent in residual function neutrophils, perhaps contributing to their milder disease phenotype [11]. However, our data show that ivacaftor treatment does induce beneficial changes in markers of activation and adhesion, consistent with neutrophils becoming less activated, and these observations were associated in some cases with significant changes in sweat chloride and lung function.

We noted a dichotomous response in terms of intra- and extracellular markers of neutrophil activation and adhesion. CD62L, CD54, CD88a and CD47 were significantly upregulated at the cell surface by ivacaftor, whereas intracellular calprotectin and CD11b decreased. CD62L and CD54 are shed upon neutrophil activation and migration [12], and an increased expression on neutrophils suggests a pool of less activated and migrating neutrophils [13]. CD88 is a receptor for C5a with lower levels being seen in critical illnesses, such as sepsis, and increases in CD88 being consistent with less inflammation and a greater potential for phagocytosis [14]. CD47 is the binding partner of signal regulatory protein and improves trans-epithelial migration of neutrophils during inflammation. Taken together this upregulation in cell surface markers would suggest a less activated circulating neutrophil pool in ivacaftor-treated patients that retains the potential to migrate and phagocytose when needed. Calprotectin (a known DAMP) and CD11b decreased significantly following treatment. Calprotectin is a major biomarker of CF in both early and late disease [15], and an association with CFTR mutations has been described in previous genetic studies. One might speculate that neutrophils containing less calprotectin would have less potential to perpetuate inflammation by releasing it as a DAMP. A decrease in CD11b expression may represent a decrease in the individual protein, or a decrease in tertiary granules where it is mostly found. When we consider that the primary granule marker CD63 did not decrease with therapy, these results suggest specific neutrophil reprogramming in response to ivacaftor.

These data highlight potential benefits of enhancing CFTR function in CF neutrophils, even in patients with CF with residual function mutations. Future work is required to assess how this relates to neutrophils from people without CF. As our experiments were performed on peripheral blood neutrophils rather than lung neutrophils, we interpret our results with a degree of caution. Our results also infer that the apoptosis defect we have previously seen in CF neutrophils is dependent on severe CFTR dysfunction, as it is absent in this patient group with residual function. Nevertheless, we show changes in neutrophil phenotype following ivacaftor treatment that suggest the development of a less inflammatory neutrophil population, which could be seen as beneficial. Further studies of immune cell function in CF patients with and without CFTR modifier therapies are now required to investigate these findings.

## Shareable PDF

10.1183/13993003.02161-2020.Shareable1 This one-page PDF can be shared freely online.
Shareable PDF ERJ-02161-2020.Shareable
