# Supplementary material for: Ivacaftor modifies cystic fibrosis neutrophil phenotype in subjects with R117H residual function CFTR mutations
Source: Eur Respir J. 2021 Jan 21;57(1):2002161. doi: 10.1183/13993003.02161-2020 (PMC7817919; doi:10.1183/13993003.02161-2020)
Supplement: Supplementary file 1 [file ERJ-02161-2020.Shareable.pdf]

AGORA
RESEARCH LETTER

SHAREABLE PDF

Ivacaftor modifies cystic fibrosis neutrophil phenotype in subjects with R117H residual function CFTR mutations

Gareth R. Hardisty[1], Sheonagh M. Law[1], Suzanne Carter[2], Brenda Grogan[2], Pradeep K. Singh[3], Edward F. McKone[2] and Robert D. Gray[1]

Affiliations: [1]University of Edinburgh, Centre for Inflammation Research, Edinburgh, UK. [2]St Vincent's Hospital, University College Dublin, Dublin, Ireland. [3]Dept of Microbiology, University of Washington School of Medicine, Seattle, WA, USA.

Correspondence: Robert D. Gray, University of Edinburgh, Centre for Inflammation Research, Queens Medical Research Centre, 47 Little France Crescent, Edinburgh, EH16 4TJ, UK. E-mail: r.d.gray@ed.ac.uk

@ERSpublications
CFTR modulation leads to changes in neutrophil phenotype even in patients with residual function CFTR mutations https://bit.ly/2EUk7xH

Cite this article as: Hardisty GR, Law SM, Carter S, *et al.* Ivacaftor modifies cystic fibrosis neutrophil phenotype in subjects with R117H residual function CFTR mutations. *Eur Respir J* 2021; 57: 2002161 [https://doi.org/10.1183/13993003.02161-2020].

This single-page version can be shared freely online.

To the Editor:

Cystic fibrosis transmembrane conductance regulator (CFTR) modulator therapy (ivacaftor, lumacaftor, tezacaftor) treats the basic defect in cystic fibrosis (CF) by increasing CFTR function and improving lung function and quality of life. CF lung disease is characterised by chronic bacterial colonisation, inflammation and excessive neutrophilia [1]. The confirmation of CFTR expression on neutrophils [2] led to speculation that immune cell dysfunction may be implicated in CF lung inflammation. Neutrophils from CF patients with severe CFTR mutations (*e.g.* F508del and G551D) have prolonged neutrophil survival [3] and decreased phagocytosis and degranulation. The residual function R117H mutation causes a 25% decrease in channel conductance [4], and when present in combination with a second severe mutation (*e.g.* F508del) results in CFTR function that lies somewhere between heathy controls and typical CF. CF patients with residual function develop disease at a later stage and ivacaftor is now licensed for the treatment of the R117H mutation having been demonstrated to be effective in clinical trials [5]. Treatment of people with G551D mutations with ivacaftor also has significant mutation specific effects on myeloid cells [6]. Therefore, we assessed the effects of CFTR modulator therapy on neutrophil phenotype and function in this group.

Copyright ©ERS 2021.. This version is distributed under the terms of the Creative Commons Attribution Licence 4.0.
